# Characteristics of Financial Exploitation in a Sample of Israeli Older Adults

**DOI:** 10.1093/geroni/igab046.2832

**Published:** 2021-12-17

**Authors:** Gali Weissberger, S Duke Han, Amit Shrira

**Affiliations:** 1 Bar Ilan University, Ramat Gan, HaMerkaz, Israel; 2 University of Southern California, Los Angeles, California, United States

## Abstract

Financial exploitation (FE) negatively affects wellbeing in older adulthood. However, characteristics of FE and its health correlates remain poorly understood. In this study, 138 Israeli older adults answered questions regarding FE history, and completed physical and mental health questionnaires. Of 138 participants, 23 reported a history of FE. FE participants were older (M birth year = 1950.35; sd = 9.65) than non-FE participants (M birth year = 1953.79; sd = 6.06; p = 0.028) and reported lower household income (p=0.001). Groups did not differ in education level or sex breakdown. The FE group reported older subjective age (p = 0.022), worse subjective cognition (p = 0.007), more depressive symptoms (p=0.002), and marginally higher anxiety symptoms (p = 0.099) than the non-FE group. Groups did not differ in reported levels of social support or number of medical conditions. When covarying for age, differences between groups in subjective cognition and depressive symptoms remained significant (ps ≤0.022), while subjective age differences became marginal (p = 0.07). The FE group responded to follow-up questions regarding FE experiences. Reported perpetrators included companies/businesses (most commonly reported, 30%), strangers, friends/neighbors, service providers, and family. Eleven reported losing 100 NIS to 10,000 NIS, and 10 reported losing 10,001 to over 100,000 NIS. Additionally, six FE participants reported that the FE is ongoing, and two reported additional FE experiences. Findings suggest that FE is related to mental and physical health of older adults. Findings also provide preliminary information regarding characteristics of FE in a sample of Israeli older adults.

